# Prospective association between 24-hour movement behaviors and mental health among overweight/obese college students: a compositional data analysis approach

**DOI:** 10.3389/fpubh.2023.1203840

**Published:** 2023-10-03

**Authors:** Shuai Wang, Wei Liang, Huiqi Song, Ning Su, Lin Zhou, Yanping Duan, Ryan E. Rhodes, Huaxuan Liu, Yi-de Yang, Wing Chung Patrick Lau, Julien Steven Baker

**Affiliations:** ^1^School of Economics and Business Administration, Chongqing University of Education, Chongqing, China; ^2^Department of Military Medical Geography, Army Medical Service Training Base, Army Medical University, Chongqing, China; ^3^School of Physical Education, Shenzhen University, Shenzhen, Guangdong, China; ^4^Department of Sport, Physical Education and Health, Hong Kong Baptist University, Kowloon, Hong Kong SAR, China; ^5^School of Physical Education, Hebei Normal University, Shijiazhuang, Hebei, China; ^6^School of Exercise Science, Physical and Health Education, University of Victoria, Victoria, BC, Canada; ^7^School of Physical Education and Sport Science, Fujian Normal University, Fuzhou, Fujian, China; ^8^School of Medicine, Hunan Normal University, Changsha, China

**Keywords:** physical activity, sedentary behavior, sleep, mental health, obesity

## Abstract

**Background:**

24-h movement behaviors, including light physical activity (LPA), moderate-to-vigorous physical activity (MVPA), screen-based sedentary behavior (SSB), non-screen-based sedentary behavior (NSB) and sleep are crucial components affecting mental health. This study aimed to examine the associations of movement behaviors with mental health outcomes among overweight/obese college students using a compositional data analysis approach.

**Methods:**

Using a prospective design, 437 Chinese college students (20.1 ± 1.7 years, 51.7% female) completed a two-wave online data collection, where demographics and movement behaviors (LPA, MVPA, SSB, NSB, sleep) were collected at baseline, while depression, anxiety and stress were measured at the 2-month follow-up (Apr-Jul 2022). Compositional data analyses were implemented using R.

**Results:**

Depression, anxiety, and stress were negatively associated with time spent in MVPA and sleep (*p* < 0.001) and positively associated with time spent in SSB and NSB (*p* < 0.001). Reallocating 15 min to MVPA from LPA, SSB and NSB predicted improvements in depression (LPA: −0.234 unit; SSB: −0.375 unit; NSB: −0.249 unit), anxiety (LPA: −0.092 unit; SSB: −0.284 unit; NSB: −0.165 unit), and stress (LPA: −0.083 unit; SSB: −0.312 unit; NSB: −0.191 unit). For dose–response relationships of 5–55 min isotemporal substitution, when time was reallocated to MVPA from LPA, NSB, and SSB, the estimated detriments to mental health were larger in magnitude than the estimated benefits of time reallocation from MVPA to LPA, NSB, and SSB.

**Conclusion:**

The findings emphasize the importance of participating in MVPA to improve mental health in overweight/obese college students during the post-COVID-19 era. The compositional analysis produced clear targets for the time allocation of these behaviors for future interventions and policymaking.

## 1. Introduction

The mental health of college students has been a global concern (1). College students are at high risk of depression and anxiety, and are exposed to multiple stressors (2). A review focusing on US undergraduates reported a prevalence depression of 22% (3). Li et al. (4) found that the pooled prevalence of depression and anxiety symptoms among college students was 33.6 and 39.0%, respectively. Mental health problems negatively affected students’ motivation, concentration, and social interaction, which in turn could hinder their academic success. Importantly, severe mental disorders (e.g., depression) are the leading causes of suicide, and bring considerable burdens to not only individuals and families but also the whole society (5).

The outbreak and continuation of the COVID-19 pandemic has resulted in massive changes in daily routines (e.g., reduced physical activity, increased sedentary time, and large-scale quarantine), subsequently increasing the risk of mental disorders among college students (6). For example, a cross-sectional survey reported that 42.8% of French university students indicated severe mental problem symptoms during the pandemic (1). Another cross-sectional study of 821,218 Chinese college students found that over half of the students had mental disorders, such as acute stress, anxiety, and depression (7). As a vulnerable population, overweight and obese college students have a higher prevalence and risk of mental health problems than those of normal weight as reported in previous studies (8). Therefore, research on mental health among college students who are overweight and obese is important, especially during the COVID-19 pandemic and beyond.

24-h movement behaviors, including physical activity (PA), sedentary behavior (SB) and sleep, have amassed considerable evidence as key correlates of mental health. Moderate-to-vigorous intensity physical activity (MVPA) is inversely associated with anxiety and depression (9). Increased sedentary time has also been significantly associated with increased levels of stress, anxiety, and depression among college students (10). Furthermore, recent research showed that college students who slept over 9 h per day had a higher score for mental disorders than those who slept 7–8 h per day (11). While PA, SB, and sleep are closely associated with mental health among college students, most studies have examined the association between PA, SB, sleep, and health outcomes in isolation (12). However, PA, SB, and sleep represent a continuous range of movement distributed throughout 24 h; these three behaviors have been referred to collectively as movement behaviors (13). All movement behaviors coexist as a whole or as components; the change in time spent on one behavior leads to changes in the time spent on other behaviors (14). Therefore, investigating the association of movement behaviors with mental health outcomes should take the codependency of these movement components into account.

The use of traditional linear regression analysis may lead to pseudo correlation and multicollinearity between component data due to the limitations of nonnegativity and summability of the composition data (15). Recently, studies have used compositional data analysis to investigate the association between movement behaviors and health outcomes, eliminating covariance between the original component data (16). For example, Mekary et al. (17) reported that the reallocation from 60 min of television watching to brisk walking was associated with a lower risk of depression in adults. A cross-sectional study of Japanese workers reported that time spent on sleeping was positively related to mental health indicators, whereas time spent on SB or LPA was negatively related (18). Additionally, a recent longitudinal study showed that reallocating 15 min from MVPA to sedentary time or LPA resulted in a significant deterioration in depressive symptoms among young adults, with 0.46 units and 0.57 units, respectively (19). However, to our knowledge, no study explored the association between 24-h movement behaviors and mental health in overweight/obese college students using compositional methods. Given the vulnerability of overweight/obese college students during the pandemic and beyond, there is a need to investigate the association between movement behaviors and mental health to inform future practice.

Therefore, this study aimed to (1) examine the prospective associations of 24-h movement behaviors, i.e., MVPA, LPA, screen-based sedentary behavior (SSB), non-screen-based sedentary behavior (NSB), and sleep with mental health outcomes (i.e., depression, anxiety, stress) among overweight/obese college students using a compositional isotemporal substitution analysis approach; and (2) investigate the dose–response relationship between movement behaviors, reallocation time, and mental health outcomes.

## 2. Methods

### 2.1. Study design, participants, procedure

This study used a two-wave prospective design. Aiming to achieve a small-to-medium effect size (Cohen *f*^2^) of 0.08 (20), with an alpha of 0.05 and 80% statistical power in the regression model, considering a dropout rate of 20% in the second-wave data collection, 194 participants were required to ensure a robust statistical analysis. Considering an overweight and obesity rate of 20% based on updated national health surveillance, a total of 970 participants were required to be recruited. Using a convenience sampling approach, we contacted 1,862 college students from four universities in China, of which 1,750 college students agreed to participate in the study (response rate: 94%). Finally, 486 college students met the inclusion criteria, including: (1) overweight/obese (i.e., BMI ≥ 24 kg/m^2^); (2) no restriction of physical mobility; (3) no sleep disorders; (4) no pre-diagnosed cognitive disabilities; (5) sufficient language skills in reading and understanding Chinese; and (6) not collegiate athletes or members of university sport teams.

All the participants were asked to sign informed consent forms before participating in the study. The demographic information and 24-h movement behaviors were collected at baseline, while the mental health indicators were measured at the 2-month follow-up. Data collection was implemented on a website platform (SOJUMP) from Apr to Jul 2022. Each survey lasted for approximately 10 min/person. Ethical approval was obtained from the Institutional Research Committee of the authors’ university. The Declaration of the Helsinki World Medical Association and the STROBE statement were used to guide the study implementation and results reporting (21).

### 2.2. Measures

#### 2.2.1. 24-h movement behaviors

Physical activity (PA) was measured using the Chinese version of the International Physical Activity Questionnaire (IPAQ-C) (ICC = 0.79) (22). Participants were invited to report the frequency (days) and duration (minutes) they spent in three intensities of PA (i.e., light PA, moderate PA, and vigorous PA) during the previous 7 days. The LPA/MVPA time (min/day) was calculated by the total LPA/MVPA time (min/week) divided by 7 days.

Screen-based sedentary behavior (SSB) and non-screen-based sedentary behavior (NSB) were measured using the Chinese translated Sedentary Behavior Questionnaire (SBQ) (ICC = 0.93) (23). Participants were asked to report the time (min) they spent in nine sedentary behaviors (e.g., watching TV, playing computer or video games, sitting and reading) on average per day. These items were categorized as SSB and NSB.

Sleep was measured using items extracted from the Chinese version of the Pittsburgh Sleep Quality Index (PSQI) (Cronbach’s alpha = 0.92) (24). Participants were asked to answer, “during the past week, how much time (hours and minutes) do you sleep on average every day?.” Sleep time was calculated using the unit of min/day to remain consistent with the unit of other movement behaviors.

#### 2.2.2. Mental health outcomes

Mental health outcomes including depression, anxiety and stress were measured using the 21-item Chinese version of the Depression Anxiety Stress Scale (DASS-21) (25). Each dimension consisted of seven items (Cronbach’s alpha = 0.79–0.89), where participants were asked to answer the question “how much the statement applied to you over the past week…,” e.g., “…I found it hard to wind down” “…I felt that I was using a lot of nervous energy.” Responses were indicated on a 4-point Likert scale, ranging from “0 = never” to “3 = always.” The total score of seven items was calculated for each dimension, with a higher score indicating more severe level of mental health problems.

#### 2.2.3. Covariates

Covariates included age, gender, grade, body mass index (BMI; kg/m^2^), yearly household income (low: <84,999 RMB; medium: 84,000–132,000 RMB; high: >132,000 RMB), and parental education level (primary or below; middle/high school; college or above) (26).

### 2.3. Data analysis

Component data analyses were performed using IBM SPSS 28.0 (Armonk, NY, United States) and R v.3.6.3 software (R Development Core Team, Vienna, Austria). Invalid and abnormal values, i.e., z-scores ≥ ± 3 standard deviation (SD) were excluded (26). All data were checked for normal distribution and homogeneity of data variance using quantile-quantile (Q-Q) plots. Descriptive statistics, including the mean, SD, and percentage (%) were used to describe the characteristics of the included participants. Standard and component descriptive statistics were calculated for comparison. The compositional mean was computed by calculating the geometric mean for each behavior (MVPA, LPA, SSB, NSB, and sleep) and then normalizing the data to the same constant as the raw data (i.e., 1). This metric is consistent with the relative and symmetric scale of the data (14). A variance matrix was used to describe the dispersion of the daily composition (15). Since the variance of individual composition data does not capture the interdependence of the movement behaviors, pairwise log-ratio variances were calculated to describe dispersion trends: when the value is close to zero, it indicates that the time spent in the two respective movement behaviors is highly proportional, while when the value is close to one, it indicates the opposite.

Multiple linear regression models were used to examine the prospective association between movement behaviors (explanatory variable) and mental health (dependent variable). The composition was represented as a set of three isometric log-ratio (*ilr*) coordinates before inclusion in the regression model (14). Covariates (age, gender, grade, income, parental education level, and BMI) were included as explanatory variables. The outcome variables were depression, anxiety, and stress. The linearity, normality, homoscedasticity, and outliers of the *ilr* multiple linear regression models were further examined.

The isotemporal reallocation analyses were conducted to predict differences in the outcome variables associated with the reallocation of fixed time durations between two movement behaviors, while the remaining behaviors were unchanged. This was achieved by creating a new series of activity compositions to simulate a 5-min reallocation between all pairs of activity behaviors, using the average composite of samples as the baseline or starting composite. The new compositions were represented as sets of *ilr* coordinates, and each composition was subtracted from the mean *ilr* coordinates to obtain the *ilr* differences. These *ilr* differences were then used to determine the estimated difference (95% CI) for all outcomes. Predictions were made for the 15-min pairwise reallocations since 15-min changes in movement behavior can significantly affect health outcomes (14). This model was repeated for the activity that had a significant effect on mental health for 15-min pairwise reallocations. Statistical significance levels were set as *p* < 0.05 (two-tailed). *R*^2^ values of 0.02, 0.13, and 0.26 indicated small, medium, and large effect sizes, respectively (27).

Sensitivity analyses were conducted to test the robustness of the study results and alternative explanations. After randomly excluding 10% of participants, all association analyses were rerun. In addition, e-values were calculated for the main findings to further estimate the plausibility of bias for unmeasured and residual confounding. The e-values estimate the strength of the unmeasured confounding factor that was required to eliminate the observed association between the independent and dependent variables, taking all measured covariates into account (28).

## 3. Results

**Figure 1:**
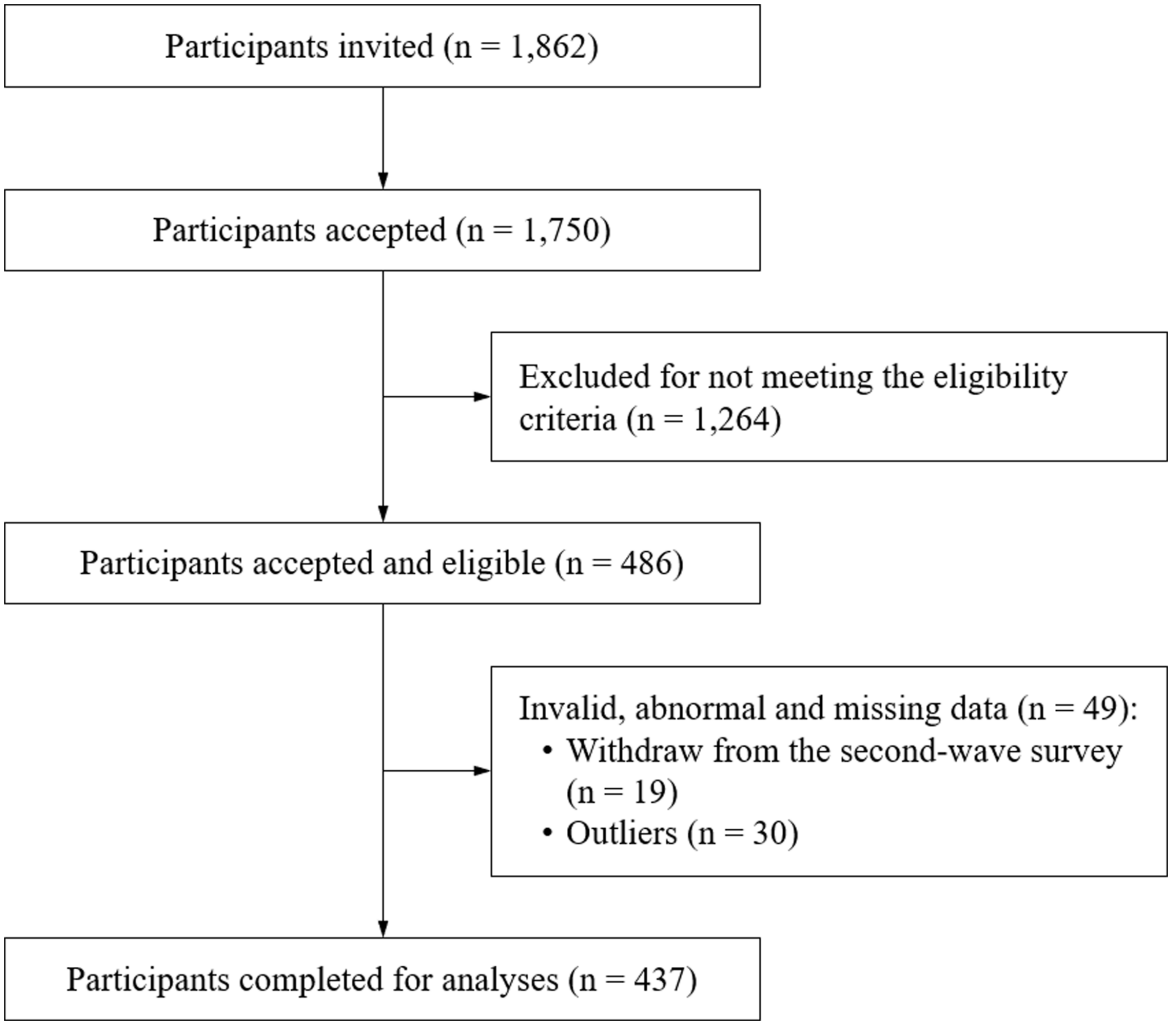
STROBE diagram of study process.

After deleting the dropouts (3.9%) and outliers (i.e., z score of standard deviation > ± 3; 6.2%), 437 eligible participants were finally included in the data analysis (Figure 1). Movement behaviors and mental health outcomes adhered to the normality distribution. The mean age of the participants was 20.1 years, and 48.3% were male. Descriptive statistics for the included participants are displayed in Table 1.

**Table 1:** Descriptive characteristics of the included participants (*n* = 437).

Variables	Mean (SD)/ *N* (%)
Age (years), mean (SD)	20.1 (1.7)
Gender, *n* (%)	
Male	211 (48.3%)
Female	226 (51.7%)
Grade, *n* (%)	
Freshman	201 (46.0%)
Sophomore	115 (26.3%)
Junior	63 (14.4%)
Senior	58 (13.3)
Height (cm), mean (SD)	169.4 (8.9)
Weight (kg), mean (SD)	78.4 (9.0)
BMI (kg/m^2^), mean (SD)	27.3 (2.5)
Yearly household income, *n* (%)	
< 84,999RMB	144 (33.0%)
84,000–132,000RMB	165 (37.8%)
> 132,000 RMB	128 (29.3%)
Parental education level, *n* (%)	
Primary or below	97 (22.2%)
Middle/high school	264 (60.4%)
College or above	76 (17.4%)
Mental health outcomes, mean (SD)	
Depression	6.0 (5.7)
Anxiety	5.9 (4.8)
Stress	6.7 (4.8)

SD, standard deviation; BMI, body mass index.

Table 2 shows the arithmetic mean and compositional means for movement behaviors. The compositional means of the participants showed that 33.1% of the 24 h were spent in sleep, 30.3% in NSB, 22.4% in SSB, 10.1% in LPA, and 4.1% in MVPA. The variability of the data is summarized in the variation matrix (Table 2). The variance of log in SSB and sleep was closest to 0 (In SSB/sleep = 0.284), reflecting that these two activities had the highest correlation. The variance of log in MVPA and SSB was largest (In MVPA/SSB = 3.768), indicating that the co-dependence between MVPA and SSB was low.

**Table 2:** Arithmetic mean, compositional mean, and variation matrix of 24-h movement behaviors (*n* = 437).

	MVPA	LPA	SSB	NSB	Sleep
Arithmetic mean (%)	105.0 (7.3)	153.1 (10.6)	323.0 (22.4)	427.9 (29.7)	431.0 (29.9)
Compositional mean (%)	59.7 (4.1)	145.0 (10.1)	323.0 (22.4)	436.0 (30.3)	476.4 (33.1)
MVPA (min/day)	0	3.458	3.768	3.316	2.799
LPA (min/day)	3.458	0	0.893	1.114	0.689
SSB (min/day)	3.768	0.893	0	0.700	0.284
NSB (min/day)	3.316	1.114	0.700	0	0.311
Sleep (min/day)	2.799	0.689	0.284	0.311	0

MVPA, moderate-to-vigorous physical activity; LPA, light physical activity; SSB, screen-based sedentary behavior; NSB, non-screen-based sedentary behavior.

The association between movement behaviors and mental health outcomes among overweight/obese university students is shown in Table 3. The results indicated that depression, anxiety, and stress were inversely associated with MVPA and sleep time and positively associated with time spent in SSB and NSB. No significant association was found between LPA time and mental health outcomes.

**Table 3:** Association between movement behaviors and overweight/obese university students’ mental health outcomes (*n* = 437).

*ilr* Regression models				Model fit	
	*B* ^1^	SE	*p* value	*R* ^2^	*p* value
Depression					
*ilr* MVPA/(LPA*NSB*SSB*Sleep)	−0.953	0.163	<0.001	0.352	<0.001
*ilr* LPA/(MVPA*NSB*SSB*Sleep)	0.489	0.307	0.111		
*ilr* SSB/(MVPA*LPA*NSB*Sleep)	4.330	0.528	<0.001		
*ilr* NSB/(MVPA*LPA*SSB*Sleep)	1.860	0.467	<0.001		
*ilr* Sleep/(MVPA*LPA *NSB*SSB)	−5.725	0.986	<0.001		
Anxiety					
*ilr* MVPA/(LPA*NSB*SSB*Sleep)	−0.547	0.138	<0.001	0.341	<0.001
*ilr* LPA/(MVPA*NSB*SSB*Sleep)	−0.185	0.260	0.477		
*ilr* SSB/(MVPA*LPA*NSB*Sleep)	4.090	0.447	<0.001		
*ilr* NSB/(MVPA*LPA*SSB*Sleep)	1.757	0.396	<0.001		
*ilr* Sleep/(MVPA*LPA *NSB*SSB)	−5.115	0.836	<0.001		
Stress					
*ilr* MVPA/(LPA*NSB*SSB*Sleep)	−0.684	0.138	<0.001	0.361	<0.001
*ilr* LPA/(MVPA*NSB*SSB*Sleep)	−0.559	0.260	0.032		
*ilr* SSB/(MVPA*LPA*NSB*Sleep)	4.097	0.447	<0.001		
*ilr* NSB/(MVPA*LPA*SSB*Sleep)	1.707	0.395	<0.001		
*ilr* Sleep/(MVPA*LPA *NSB*SSB)	−4.560	0.835	<0.001		

MVPA, moderate-to-vigorous physical activity; LPA, light physical activity; SSB, screen-based sedentary behavior; NSB, non-screen-based sedentary behavior; B1, unstandardized regression coefficient of the first ilr coordinate, referring to the association of time spent in one specific behavior relative to time spent in other movement components on mental health, e.g., ilr MVPA/(LPA*NSB*SSB)*Sleep denotes the association of MVPA time relative to LPA, NSB, SSB and sleep time on mental health outcomes; SE, standard error.

Table 4 presents the estimated differences in mental health outcomes for reallocations of 15 min between movement behaviors. Reallocating 15 min to MVPA from other behaviors (except sleep) predicted improvements in depression (with LPA: −0.323 to −0.155 units; with SSB: −0.437 to −0.314 units; with NSB: −0.315 to −0.184 units), anxiety (with LPA: −0.163 to −0.021 units; with SSB: −0.336 to −0.231 units; with NSB: −0.220 to −0.110 units), and stress (with LPA: −0.153 to −0.012 units; with SSB: −0.364 to −0.259 units; with NSB: −0.246 to −0.136 units).

**Table 4:** Association of movement behaviors 15 min/day isotemporal substitution with mental health outcomes (n = 437).

	MVPA↑	LPA↑	SSB↑	NSB↑	Sleep↑
	*Est.* (95%CI)	*Est.* (95%CI)	*Est.* (95%CI)	*Est.* (95%CI)	*Est.* (95%CI)
Depression					
MVPA↓	N/A	−0.239 (−0.323, −0.155)^*^	−0.375 (−0.437, −0.314)^*^	−0.249 (−0.315, −0.184)^*^	−0.027 (−0.131, 0.077)
LPA↓	0.290 (0.195, 0.384)^*^	N/A	−0.141 (−0.203, −0.079)^*^	−0.015 (−0.071, 0.041)	0.207 (0.113, 0.302)^*^
SSB↓	0.423 (0.346, 0.499)^*^	0.128 (0.062, 0.194)^*^	N/A	0.118 (0.074, 0.162)^*^	0.340 (0.247, 0.432)^*^
NSB↓	0.303 (0.221, 0.385)^*^	0.008 (−0.052, 0.069)	−0.128 (−0.173, −0.083)^*^	N/A	0.220 (0.142, 0.298)^*^
Sleep↓	0.088 (−0.032, 0.208)	−0.207 (−0.304, −0.109)^*^	−0.343 (−0.436, −0.250)^*^	−0.217 (−0.294, −0.140)^*^	N/A
Anxiety					
MVPA↓	N/A	−0.092 (−0.163, −0.021)^*^	−0.284 (−0.336, −0.231)^*^	−0.165 (−0.220, −0.110)^*^	0.037 (−0.051, 0.125)
LPA↓	0.125 (0.045, 0.206)^*^	N/A	−0.190 (−0.243, −0.137)^*^	−0.071 (−0.119, −0.024)^*^	0.130 (0.050, 0.210)*
SSB↓	0.308 (0.243, 0.373)^*^	0.184 (0.128, 0.240)^*^	N/A	0.111 (0.074, 0.148)^*^	0.312 (0.234, 0.391)*
NSB↓	0.195 (0.125, 0.265)^*^	0.071 (0.020, 0.122)^*^	−0.121 (−0.159, −0.082)^*^	N/A	0.200 (0.134, 0.265)*
Sleep↓	0.000 (−0.101, 0.101)	−0.124 (−0.207, −0.041)^*^	−0.316 (−0.394, −0.237)^*^	−0.197 (−0.262, −0.132)^*^	N/A
Stress					
MVPA↓	N/A	−0.083 (−0.153, −0.012)^*^	−0.312 (−0.364, −0.259)^*^	−0.191 (−0.246, −0.136)^*^	−0.007 (−0.095, 0.081)
LPA↓	0.128 (0.048, 0.208)^*^	N/A	−0.223 (−0.276, −0.171)^*^	−0.103 (−0.150, −0.055)^*^	0.081 (0.001, 0.161)*
SSB↓	0.344 (0.279, 0.408)^*^	0.221 (0.165, 0.277)^*^	N/A	0.113 (0.076, 0.150)^*^	0.297 (0.219, 0.375)*
NSB↓	0.229 (0.159, 0.299)^*^	0.106 (0.055, 0.157)^*^	−0.123 (−0.161, −0.084)^*^	N/A	0.182 (0.116, 0.248)*
Sleep↓	0.051 (−0.050, 0.152)	−0.072 (−0.155, 0.011)	−0.301 (−0.379, −0.222)^*^	−0.180 (−0.245, −0.115)^*^	N/A

Estimation of change in depression, anxiety or stress when the behavior in the rows substitutes the behavior in the columns; ↑time increase; ↓time decrease; N/A, not applicable; MVPA, moderate-to-vigorous physical activity; LPA, light physical activity; SSB, screen-based sedentary behavior; NSB, non-screen-based sedentary behavior; CI, confidence interval; ^*^p < 0.05.

Reallocating 15 min from LPA, SSB, and NSB to sleep was associated with predicted decreases in depression (0.207 units, 0.343 units, 0.217 units, respectively), anxiety (0.124 units, 0.316 units, 0.197 units, respectively), and stress (0.072 units, 0.301 units, 0.180 units, respectively). The reallocations of 15 min from LPA to SSB showed significantly lower predicted 0.141 units changes in depression. The reallocations of 15 min from LPA to SSB or NSB yielded significantly lower predicted changes in anxiety (0.190 units and 0.071 units, respectively) and stress (0.223 units and 0.103 units, respectively). When 15 min were reallocated from SSB to NSB, depression, anxiety, and stress were predicted to be 0.118, 0.111, and 0.113 units higher, respectively.

**Figure 2:**
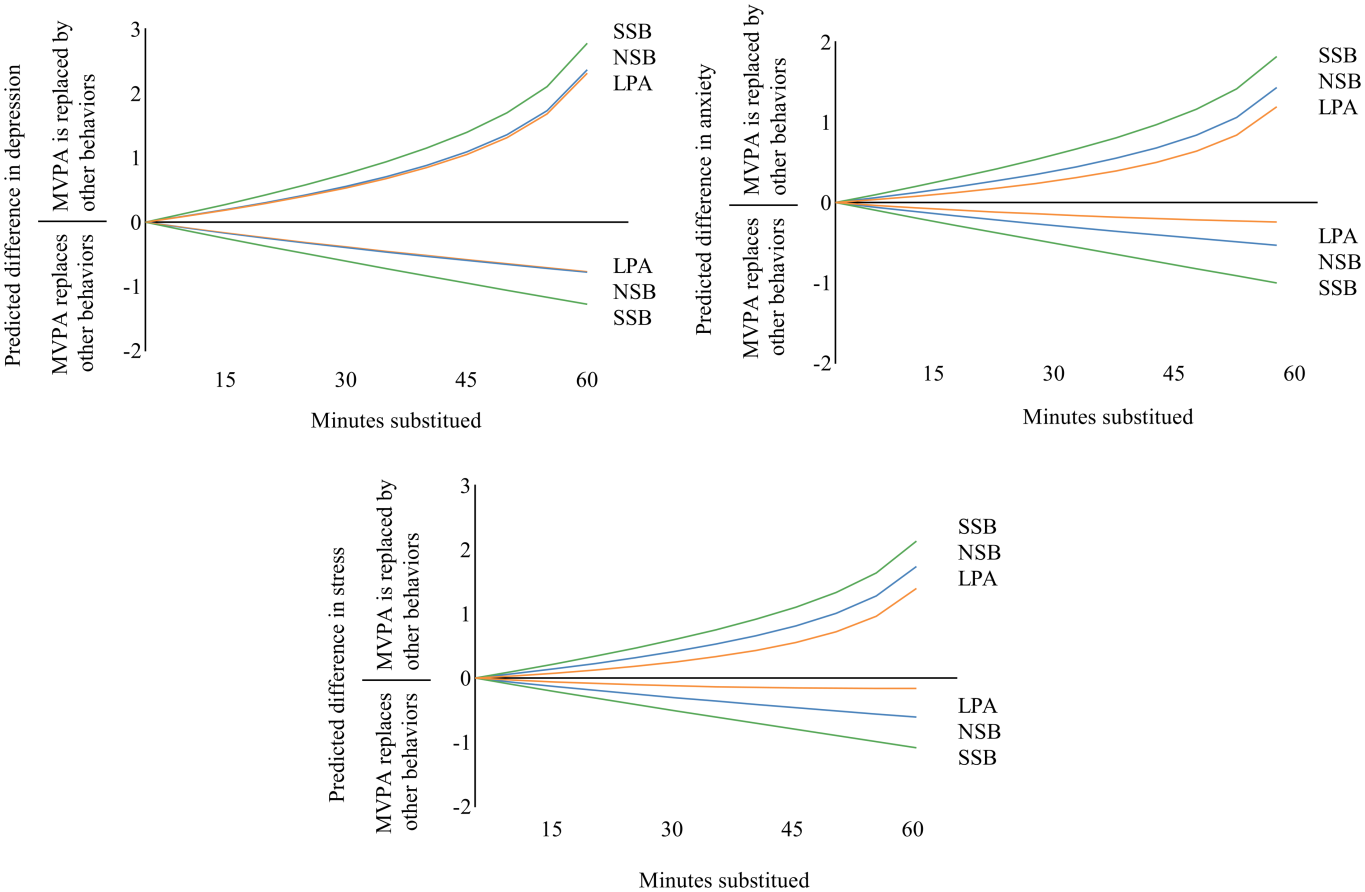
Estimated changes (95%CI) in depression, anxiety and stress for 5-55-min isotemporal substitution between SB and other movement behaviors. MVPA, moderate-to-vigorous physical activity; LPA, light physical activity; SSB, screen-based sedentary behavior; NSB, non-screen-based sedentary behavior.

For the movement behaviors with significant alterations, since the compositional mean of MVPA was 59.7 min in this study, the dose–response of mutual reallocation of movement behaviors and mental health was explored in increments of 5 min and continued for longer periods of up to 55 min to facilitate comparison with previous studies (29). The reallocations from MVPA to SSB showed the greatest differences in all predicted mental health outcomes. When MVPA was replaced by other behaviors, the nonlinearity of predicted mental health outcomes over increasing time reallocation was particularly obvious. These associations were asymmetric. The benefits of increasing time in MVPA at the cost of SSB, NSB, and sleep were lower than the negative effects of reallocating time away from MVPA to SSB, NSB, and sleep (Figure 2).

The sensitivity analyses were conducted with a sample of 393 (90% of the full sample) participants after randomly eliminating 10% (44/437) of the participants. The results were consistent with the main findings from the full sample. The e-values showed that the main findings could not be overturned by undetected covariates (see Supplementary Material S1).

## 4. Discussion

To the best of our knowledge, this is the first study to employ compositional data analysis in overweight/obese college students that accounted for the association between MVPA, LPA, SSB, NSB, and sleep in a 24-h cycle and mental health during the post-COVID-19 era. The main findings showed that replacing LPA, SSB, and NSB with MVPA predicted higher mental health. Reallocating 15 min from LPA, SSB, and NSB to sleep was associated with predicted decreases in mental health. The reallocation between MVPA, LPA, SSB, and NSB had asymmetric effects on mental health outcomes.

In this study, overweight/obese college students spent 33.1% of their 24 h in sleep, 30.0% in NSB, 22.4% in SSB, 10.1 in LPA, and 4.1% in MVPA. Since there is scarce evidence on 24-h movement behaviors among young adults who are overweight or obese, we are not able to make comparisons with the same population. However, compared to the general young adults in previous studies, our study participants exhibited prolonged periods of sedentary behavior while displaying shorter episodes of LPA and MVPA (19, 30). Arigo et al. (31) discovered that impulsivity poses a risk to decreasing MVPA among college students with obesity risk (31). As obese students tend to have higher levels of impulsivity and are susceptible to the instant gratification of sedentary habits at the expense of adhering to MVPA for longer periods (32), these findings serve as a reminder to prioritize the reduction of sedentary behavior and the promotion of MVPA among obese college students in China.

This study found that the reallocations from LPA, SSB, and NSB to MVPA were significantly associated with lower depression, anxiety, and stress symptom scores, which is consistent with previous studies with college students (19, 33). Adults showed a downwards trend in PA levels due to pandemic-related restrictions (34). Weinstein et al. (35) found that preventing people from participating in PA was related to an increase in depression and anxiety symptoms, and this increase was greater when withdrawal lasted more than 2 weeks. From a neurobiological perspective, MVPA may have a positive impact on brain structure and function. MVPA can increase BDNF levels in the central nervous system, improving anxiety and depressive symptoms (36). The increased dopamine, serotonin, and noradrenaline concentrations in the brain during participation in PA prevent the onset of mental disorders (37). The findings strongly support the need to promote MVPA after COVID-19, which may have profound effects on sustaining mental health in college students. In addition, VanKim and Nelson (38) found that socializing mediated the relationships between vigorous PA and mental health, which suggests that the positive benefits of MVPA on mental health may be achieved through the social pathway. Future studies could investigate potential moderators to enhance mental health.

Furthermore, this study found that replacing LPA, SSB, and NSB with sleep predicted lower mental disorder symptom scores. This finding is in accordance with a previous study among older adults, which reported that replacing 60 min of SB with sleep was associated with lower anxiety and depression symptom scores (39). However, a review of isotemporal substitution studies pointed out that most studies have not accounted for the reallocation between sleep time and other behaviors (20). Sleep deprivation can directly result in daytime sleepiness and unsuccessful mood regulation strategies, leading to more negative emotions and an increased risk of mental illness (40). Given the codependence of sleep with SB and PA, future studies should include sleep in isochronous substitution models.

The benefits to mental health by the reallocation of time from LPA, SSB, and NSB to MVPA were smaller in magnitude than the negative effects of time reallocation from MVPA to LPA, SSB, and NSB, which is consistent with previous studies with children and adults (29, 41). The relative contributions of 24-h movement behavior compositions partially explain the asymmetry of this association. For instance, the time spent on MVPA was only a small percentage of the total 24 h (4.1% in the present study), which is much greater than the time spent on NSB, contributing the majority of the 24-h (30.3% in the present study) (42). Another possible explanation is that the physiological adaptation associated with overloading exercise capacity (for example, participating in more MVPA) occurs slowly, leading to smaller improvements in mental health. On the other hand, the effects of detraining and reversibility (for example, increased SB) occur more quickly and may negatively affect health outcomes to a greater extent (13). This finding suggests that overweight/obese college students should maintain or increase MVPA participation to avoid significant adverse effects on mental health.

The dose–response of reallocation of movement behaviors and mental health showed that the largest decreases in depression, anxiety, and stress symptom scores occurred when MVPA replaced SSB. The change in predicted mental health scores was stronger for the MVPA alternative to SSB compared to the LPA. The difference in energy consumption between MVPA and SSB is greater than that between MVPA and LPA; thus, there is a higher probability of interchangeability between MVPA and SSB to enhance mental health. The effect of MVPA replacing SSB is stronger than that of MVPA replacing NSB on mental health, which may be explained by the varying mental health effects of mentally active and mentally passive SB (34). Hallgren et al. (43) suggested that the negative mood states associated with passive SB (e.g., SSB) may be more likely to heighten the risk of depression than mentally active SB, despite equivalent energy expenditure. During the pandemic, gyms and other recreational facilities in universities and public institutions were closed and distance learning replaced offline learning, leading to an increase in SSB and decrease in MVPA among university students (44). The government and universities should open gyms appropriately and gradually restore offline learning to lessen mental health problem symptoms among college students.

The main strength of this study is the use of compositional and isotemporal approaches to assess the association between movement behaviors and mental health in overweight/obese college students. However, this study also has some limitations. First, due to the nonrandom sampling approach, the representativeness and generalizability of the study findings are problematic. A stratified random sampling approach should be applied in future studies. Second, the use of self-reported movement behaviors and mental health outcomes, although necessary to identify the types of activities, may result in the vulnerable recall of results and social desirability bias. Moreover, poor sleep quality is associated with mental health problems (45). This study only measured sleep duration, and sleep quality needs to be considered in future studies. Additionally, it is important to note that a series of psychosocial factors (e.g., motivational and volitional factors) that may influence the change in movement behaviors, were not considered in this study (46). Future research should take these psychosocial confounders into account when examining the impacts of movement behaviors on health outcomes.

## 5. Conclusion

This study identified a negative association of MVPA and sleep and a positive association of SSB and NSB with depression, anxiety and stress among Chinese college students who were overweight or obese. Replacing 15 min of MVPA with other movement behaviors predicted improvements in all mental health indicators. In addition, for the dose–response relationship of 5–60 min isotemporal substitution, when time was reallocated to MVPA from other behaviors, the estimated detriments to mental health were larger in magnitude than the estimated benefits of time reallocation from MVPA to other behaviors. The research findings add timely evidence related to 24-h movement behaviors and mental health during the post-COVID-19 era. The findings presented here contribute to future interventions and policymaking on promoting mental health among overweight and obese college students.

## Data availability statement

The raw data supporting the conclusions of this article will be made available by the authors, without undue reservation.

## Ethics statement

The studies involving humans were approved by Institutional Research Committee of Hebei Normal University. The studies were conducted in accordance with the local legislation and institutional requirements. The participants provided their written informed consent to participate in this study.

## Author contributions

WL, SW, and HS: design and methodology, data screening and analysis. WL, NS, HL, Y-dY, and LZ: investigation and data management. WL, SW and HS: first draft writing. NS, RR, YD, JB, Y-dY, and WL: manuscript revision. All authors contributed to the article and approved the submitted version.
