# Supplementary material for: Prospective association between 24-hour movement behaviors and mental health among overweight/obese college students: a compositional data analysis approach
Source: Front Public Health. 2023 Oct 3;11:1203840. doi: 10.3389/fpubh.2023.1203840 (PMC10579788; doi:10.3389/fpubh.2023.1203840)
Supplement: Supplementary file 1 [file Table_1.DOCX]

Supplementary Material

Prospective Association between 24-Hour Movement Behaviors and Mental Health among Overweight/Obese College Students: A Compositional Data Analysis Approach

Shuai Wang^#^, Wei Liang^#^, Huiqi Song^*^, Ning Su, Lin Zhou, Yanping Duan, Ryan E. Rhodes, Huaxuan Liu, Yi-de Yang, Wing Chung Patrick Lau, Julien Steven Baker

**# Co-first authors**

*** Correspondence:**Huiqi Song
19481772@life.hkbu.edu.hk

Table S1. Sensitivity analyses for the association of movement behaviors with overweight/obese university students’ mental health outcomes (n = 393)

| ilr Regression models |  | | | Model fit | |
| --- | --- | --- | --- | --- | --- |
|  | B1 | SE | p value | *R*^2^ | *p* value |
| Depression | | | | | |
| ilr MVPA/(LPA*NSB*SSB*Sleep) | -0.949 | 0.177 | <0.001 | 0.343 | <0.001 |
| ilr LPA/(MVPA*NSB*SSB*Sleep) | 0.508 | 0.344 | 0.141 |  |  |
| ilr NSB/(MVPA*LPA*SSB*Sleep) | 1.737 | 0.494 | <0.001 |  |  |
| ilr SSB/(MVPA*LPA*NSB*Sleep) | 4.232 | 0.568 | <0.001 |  |  |
| ilr Sleep/(MVPA*LPA *NSB*SSB) | -5.529 | 1.083 | <0.001 |  |  |
| Anxiety | | | | | |
| ilr MVPA/(LPA*NSB*SSB*Sleep) | -0.569 | 0.147 | <0.001 | 0.341 | <0.001 |
| ilr LPA/(MVPA*NSB*SSB*Sleep) | -0.079 | 0.286 | 0.782 |  |  |
| ilr NSB/(MVPA*LPA*SSB*Sleep) | 1.726 | 0.410 | <0.001 |  |  |
| ilr SSB/(MVPA*LPA*NSB*Sleep) | 3.950 | 0.471 | <0.001 |  |  |
| ilr Sleep/(MVPA*LPA *NSB*SSB) | -5.027 | 0.898 | <0.001 |  |  |
| Stress | | | | | |
| ilr MVPA/(LPA*NSB*SSB*Sleep) | -0.645 | 0.148 | <0.001 | 0.3494 | <0.001 |
| ilr LPA/(MVPA*NSB*SSB*Sleep) | -0.455 | 0.288 | 0.115 |  |  |
| ilr NSB/(MVPA*LPA*SSB*Sleep) | 1.736 | 0.413 | <0.001 |  |  |
| ilr SSB/(MVPA*LPA*NSB*Sleep) | 4.080 | 0.471 | <0.001 |  |  |
| ilr Sleep/(MVPA*LPA *NSB*SSB) | -4.716 | 0.905 | <0.001 |  |  |

Table S2. Sensitivity analyses for the association of movement behavior 15 min/day isotemporal substitution with mental health (n = 393)

|  | LPA | MVPA | SSB | NSB | Sleep |
| --- | --- | --- | --- | --- | --- |
| Depression | | | | | |
| LPA |  | 0.281 (0.182, 0.381)* | -0.135 (-0.202, -0.068)* | -0.010 (-0.071, 0.050) | 0.203 (0.098, 0.309)* |
| MVPA | -0.234 (-0.323, -0.145)* |  | -0.364 (-0.429, -0.300)* | -0.240 (-0.308, -0.171)* | -0.026 (-0.138, 0.086) |
| SSB | 0.122 (0.052, 0.193)* | 0.408 (0.329, 0.488)* |  | 0.117 (0.070, 0.163)* | 0.330 (0.229, 0.431)* |
| NSB | 0.004 (-0.062, 0.070) | 0.290 (0.204, 0.376)* | -0.127 (-0.174, -0.079)* |  | 0.212 (0.127, 0.297)* |
| Sleep | -0.203 (-0.313, -0.094)* | 0.083 (-0.045, 0.211) | -0.333 (-0.435, -0.232)* | -0.209 (-0.293, -0.125)* |  |
| Anxiety | | | | | |
| LPA |  | 0.135 (0.053, 0.218)* | -0.174 (-0.230, -0.119)* | -0.061 (-0.112, -0.011)* | 0.138 (0.050, 0.225)* |
| MVPA | -0.103 (-0.177, -0.030)* |  | -0.278 (-0.332, -0.225)* | -0.165 (-0.222, -0.108)* | 0.034 (-0.060, 0.127) |
| SSB | 0.167 (0.109, 0.226)* | 0.302 (0.236, 0.368)* |  | 0.106 (0.067, 0.144)* | 0.304 (0.221, 0.388)* |
| NSB | 0.060 (0.005, 0.115)* | 0.195 (0.123, 0.266)* | -0.115 (-0.155, -0.075)* |  | 0.197 (0.127, 0.267)* |
| Sleep | -0.132 (-0.223, -0.042)* | 0.002 (-0.104, 0.108) | -0.308 (-0.392, -0.223)* | -0.194 (-0.264, -0.125)* |  |
| Stress | | | | | |
| LPA |  | 0.122 (0.038, 0.205)* | -0.213 (-0.268, -0.157)* | -0.094 (-0.145, -0.043)* | 0.096 (0.008, 0.184)* |
| MVPA | -0.082 (-0.156, -0.008)* |  | -0.299 (-0.353, -0.245)* | -0.180 (-0.238, -0.123)* | -0.010 (-0.084, 0.104) |
| SSB | 0.209 (0.150, 0.268)* | 0.326 (0.260, 0.393)* |  | 0.110 (0.072, 0.149)* | 0.301 (0.216, 0.385)* |
| NSB | 0.106 (0.055, 0.157)* | 0.229 (0.159, 0.299)* | -0.123 (-0.161, -0.084)* |  | 0.182 (0.116, 0.248)* |
| Sleep | -0.088 (-0.179, 0.004) | 0.030 (-0.077, 0.137) | -0.304 (-0.389, -0.220)* | -0.186 (-0.256, -0.116)* |  |

Estimation of change in depression, anxiety or stress when the behavior in the rows substitutes the behavior in the columns

Table S3. E-values for the association of movement behaviors with overweight/obese university students’ mental health outcomes (n = 437)

|  | Depression | Anxiety | Stress |
| --- | --- | --- | --- |
| *ilr* MVPA/(LPA*NSB*SSB*Sleep) | 1.71 (1.52) | 1.53 (1.33) | 1.63 (1.44) |
| *ilr* LPA/(MVPA*NSB*SSB*Sleep) | 1.44 (1) | 1.26 (1) | 1.54 (1.12) |
| *ilr* NSB/(MVPA*LPA*SSB*Sleep) | 2.25 (1.71) | 2.39 (1.83) | 2.35 (1.8) |
| *ilr* SSB/(MVPA*LPA*NSB*Sleep) | 4.16 (3.26) | 4.66 (3.67) | 4.67 (3.68) |
| *ilr* Sleep/(MVPA*LPA *NSB*SSB) | 5.69 (3.67) | 6.1 (3.95) | 5.28 (3.39) |

Table S4. E-values for the association of movement behavior 15 min/day isotemporal substitution with mental health (n = 437)

|  | LPA | MVPA | SSB | NSB | Sleep |
| --- | --- | --- | --- | --- | --- |
| Depression | | | | | |
| LPA |  | 1.93 (1) | 1.53 (1) | 1.13 (1) | 1.71 (1) |
| MVPA | 1.79 (1) |  | 2.16 (1.29) | 1.82 (1) | 1.18 (1) |
| SSB | 1.5 (1) | 2.3 (1) |  | 1.47 (1) | 2.07 (1) |
| NSB | 1.09 (1) | 1.96 (1) | 1.5 (1) |  | 1.74 (1) |
| Sleep | 1.71 (1) | 1.38 (1) | 2.07 (1) | 1.73 (1) |  |
| Anxiety | | | | | |
| LPA |  | 1.49 (1) | 1.66 (1) | 1.33 (1) | 1.5 (1) |
| MVPA | 1.4 (1) |  | 1.91 (1.13) | 1.6 (1) | 1.22 (1) |
| SSB | 1.65 (1) | 1.98 (1) |  | 1.45 (1) | 1.99 (1) |
| NSB | 1.33 (1) | 1.68 (1) | 1.48 (1) |  | 1.69 (1) |
| Sleep | 1.49 (1) | 1 (1) | 2 (1) | 1.68 (1) |  |
| Stress | | | | | |
| LPA |  | 1.5 (1) | 1.75 (1) | 1.43 (1) | 1.36 (1) |
| MVPA | 1.37 (1) |  | 1.99 (1.24) | 1.67 (1) | 1.09 (1) |
| SSB | 1.74 (1) | 2.08 (1) |  | 1.45 (1) | 1.95 (1) |
| NSB | 1.44 (1) | 1.77 (1) | 1.48 (1) |  | 1.64 (1) |
| Sleep | 1.34 (1) | 1.27 (1) | 1.96 (1) | 1.64 (1) |  |

Estimation of change in depression, anxiety or stress when the behavior in the rows substitutes the behavior in the columns
